# Sphingosine kinases negatively regulate the expression of matrix metalloproteases (*MMP1* and *MMP3*) and their inhibitor *TIMP3* genes via sphingosine 1‐phosphate in extravillous trophoblasts

**DOI:** 10.1002/rmb2.12379

**Published:** 2021-03-22

**Authors:** Kirti R. Chahar, Vijay Kumar, Phulwanti K. Sharma, Daniela Brünnert, Vibha Kaushik, Pragya Gehlot, Indu Shekhawat, Suman Kumar, Ajay Kumar Sharma, Sandhya Kumari, Pankaj Goyal

**Affiliations:** ^1^ Department of Biotechnology School of Life Sciences Central University of Rajasthan Ajmer India; ^2^ Comprehensive Cancer Center Mainfranken Translational Oncology University Hospital of Würzburg Würzburg Germany; ^3^ Department of Obstetrics & Gynecology J. L. N. Medical College Ajmer India

**Keywords:** extravillous trophoblast, matrix metalloproteinases, Sphingosine 1‐phosphate, Sphingosine kinase, tissue inhibitors of metalloproteases

## Abstract

**Purpose:**

Extracellular matrix remodeling is essential for extravillous trophoblast (EVT) cell migration and invasion during placental development and regulated by matrix metalloproteinases (MMPs) and tissue inhibitors of metalloproteases (TIMPs). Sphingosine kinases (SPHK1 and SPHK2) synthesize sphingosine‐1‐phosphate (S1P), which works either intracellularly or extracellularly via its receptors S1PR1‐5 in an autocrine or paracrine manner. The role of SPHKs/S1P in regulating the expression of MMPs and TIMPs in EVT is mostly unknown and forms the primary objective of the study.

**Methods:**

HTR‐8/SVneo cells were used as a model of EVT. To inhibit the expression of SPHKs, cells were treated with specific inhibitors, SK1‐I and SKI‐II, or gene‐specific siRNAs. The expressions of *MMPs and TIMPs* were estimated by qPCR.

**Results:**

We demonstrated that *SPHK1*, *MMP1‐3*, and *TIMP1‐3* were highly expressed in HTR‐8/SVneo cells. We found that treatment of cells with SK1‐I, SKI‐II, and knockdown of *SPHK1* or *SPHK2* increased the expression of *MMP1*, *MMP3*, and *TIMP3*. The addition of extracellular S1P inhibits the upregulation of *MMPs* and *TIMPs* in treated cells.

**Conclusions:**

SPHKs negatively regulate the expression of *MMP1*, *MMP3*, and *TIMP3*. The level of intracellular S1P acts as a negative feedback switch for *MMP1*, *MMP3*, and *TIMP3* expression in EVT cells.

## Graphical abstract

The levels of intracellular S1P act as a controlling switch for *MMP1*, *MMP3*, and *TIMP3* expression in EVT cells suggesting a new role of intracellular S1P in ECM remodeling.

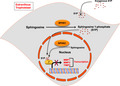

## 1 INTRODUCTION

Extravillous trophoblast (EVT) cell migration is crucial during placental development, spiral artery remodeling, and successful pregnancy outcome. Aberrant cell migration causes various pregnancy‐related diseases, such as preeclampsia^1^ and placental abnormalities.^2^ Extracellular matrix (ECM) remodeling is one of the critical processes for proper cell migration. It is tightly regulated by ECM‐degrading enzymes, such as matrix metalloproteases (MMPs) and tissue inhibitors of metalloproteases (TIMPs) family proteins.^3^

MMPs are zinc‐dependent proteases that are secretory or membrane proteins and get activated during matrix remodeling in various physiological and pathophysiological conditions.^3^ There are 24 MMP genes reported in humans that encode for 23 MMP proteins.^3^, ^4^ To avoid excessive and deleterious degradation of tissues, TIMPs inhibit activated‐MMPs and maintain tissue homeostasis. TIMP family consists of four members, TIMP1, TIMP2, TIMP3, and TIMP4. Except for TIMP3, all TIMPs are secretory proteins.^5^ Co‐expression of MMPs and TIMPs has been demonstrated in trophoblast cells.^6^ MMP2 and MMP7 have a significant role in endometrial tissue remodeling during decidualization.^7^, ^8^ Various studies showed that nearly all MMPs were expressed in decidua and cytotrophoblasts.^6^, ^9^, ^10^, ^11^

Sphingosine‐1‐phosphate (S1P), a sphingolipid, plays a vital role in various cellular processes, including cell proliferation, cell migration, secretion, and apoptosis.^12^, ^13^, ^14^ S1P is synthesized intracellularly by the phosphorylation of sphingosine through two conserved Sphingosine kinases (SPHK1 and SPHK2).^15^ S1P works either intracellularly as a secondary messenger or through its receptors S1PR1‐5 in an autocrine or paracrine manner.^16^ Intracellular S1P can regulate the expression of various genes by binding to many intracellular targets, such as histone deacetylase (HDAC),^17^ atypical protein kinase C (aPKC),^18^ PPARγ,^19^ and TNF receptor‐associated factor 2 (TRAF2).^20^

S1PR1/S1P signaling is required for the expression of MMP2 in bone marrow‐derived mesenchymal stromal cells.^21^ The expression of MMP2 and MMP9 was upregulated by S1P treatment in pancreatic cancer cells.^22^ S1P promotes EVT cell invasion through MMP2 using S1P/S1PR1 signaling.^23^ Knockdown of SPHK1 inhibited the secretion of MMP2 and MMP9 in fibroblast‐like synoviocytes.^24^ Recently, Liu et al. showed that SPHK1 promotes MMP2 and MMP9 expression in the colon cancer cell line RKO.^25^ A recent study showed that S1P inhibits migration of chondrosarcoma through upregulation of TIMP3 expression.^26^ TIMP3 was upregulated in HDAC9‐knockdown HTR‐8/SVneo cells.^27^ SPHK1 is activated via TGF‐β and mediates TIMP1 upregulation.^28^

In the present study, we investigated whether SPHK activity and intracellular S1P play any role in the expression of MMPs and TIMPs in EVT cells. Indeed, we found that the expression of *MMP1*, *MMP3*, and *TIMP3* genes was regulated through the intracellular level of S1P synthesized by SPHK1 and SPHK2.

## 2 MATERIAL AND METHODS

### 2.1 Materials

Primers were synthesized from Eurofins, India, and IDT, India. SPHK1 inhibitor SK1‐I was purchased from Enzo Life Sciences, USA. S1P and SKI‐II were procured from Cayman chemicals. TAMRA‐S1P was from Echelon Biosciences. All cell culture reagents were purchased from HyClone, GE Life Sciences. iScript cDNA synthesis kit was from Bio‐Rad and DyNAmo ColorFlash SYBR Green qPCR kit was from Thermo Fisher. Control, *SPHK1,* and *SPHK2* specific siRNAs were procured from Sigma‐Aldrich.

### 2.2 Cell culture

HTR‐8/SVneo cell line, a first‐trimester human EVT cell line, was a kind gift from Dr Charles H Graham.^29^ These cells were grown as described previously.^12^ Cells were grown in factor‐reduced media (basal media + 5% charcoal‐stripped FBS [dFBS]) 24 hours prior to the experiment. Cells were kept in serum‐starved media (basal media + 0.5% dFBS) and treated with inhibitors for 30 minutes before activating with S1P for the specified time.

### 2.3 Real‐time PCR

Cells were lysed with TRIzol reagent (Invitrogen), and then total RNA was isolated as per the manufacturer's protocol. cDNA was synthesized with an iScript cDNA synthesis kit as per the manufacturer's protocol. Primers were designed using the NCBI Primer‐BLAST tool, and real‐time PCR was performed on Roche LightCycler 96 machine, as described previously.^30^ The list of primers used in this study are shown in Table S1. The cycling program followed for the reaction included 7 minutes of initial denaturation at 95°C and then 40 cycles with 10 seconds at 95°C and 20 seconds at 60°C. The specificity of the amplicons was analyzed by thermal dissociation curve and agarose gel electrophoresis. Data were normalized against the house‐keeping gene, *β‐actin*.

### 2.4 Gene silencing

Fifty thousand cells/well were grown in 24‐well plates. Transfection of *SPHK1* and *SPHK2* specific siRNAs was performed in duplicate wells using Lipofectamine RNAiMAX transfection reagent as per manufacturer's protocol. siRNA (10 pmol) was added to each well, and the plates were incubated for 48‐72 hours with 5% CO_2_ in a humidified incubator at 37°C. Scrambled siRNA was used as a negative control.

### 2.5 Immunofluorescent staining and fluorescence microscopy

HTR‐8/SVneo cells were grown in a 24‐well plate and incubated with TAMRA‐S1P (1 μM) for 1 hour. For nuclear staining, Hoechst 33258 was added to the cell culture media for 20 minutes before capturing the images. After incubation, cells were washed with PBS, and images were taken with Zeiss Axio Observer fluorescence microscope with ZEN software.

### 2.6 Gelatin zymography

Expression of MMP1 and MMP3 in the HTR‐8/SVneo cells was analyzed using gelatin zymography as described previously with modifications.^31^ Cells were grown in a 12‐well plate and were treated with S1P, SK1‐I, and SKI‐II as described earlier. Control and treated cells were lysed in zymogen sample buffer (62.5 mM Tris pH 6.8, 10% v/v glycerol, 2% SDS, 0.01% w/v bromophenol blue). The cell lysates were subjected to 10% SDS‐PAGE (without β‐mercaptoethanol) containing 1 mg/mL gelatin. The gel was briefly washed with distilled water and kept in a renaturation buffer (2.5% Triton X‐100) for 1 hour at room temperature. The gel was then incubated in development buffer (50 mM Tris‐HCl pH 7.5, 200 mM NaCl, 5 mM CaCl_2_ and 0.02% Tween‐20) overnight at 37°C. The gel was stained with 0.1% (w/v) coomassie brilliant blue R‐250 and destained with deionized water.

### 2.7 Statistical analysis

Every experiment was performed three or more times independently with different passages of cells. Statistical analysis was performed using GraphPad Prism 7 software (GraphPad, La Jolla, CA, USA) using one‐way ANOVA or two‐tailed unpaired *t* test. The results are calculated as mean ± SD and data are shown as mean + SD. *P*‐values of <.05 were considered statistically significant.

## 3 RESULTS

### 3.1 Expression profiling of *SPHK*, *MMP*, and *TIMP* genes in HTR‐8/SVneo cells

**FIGURE 1:**
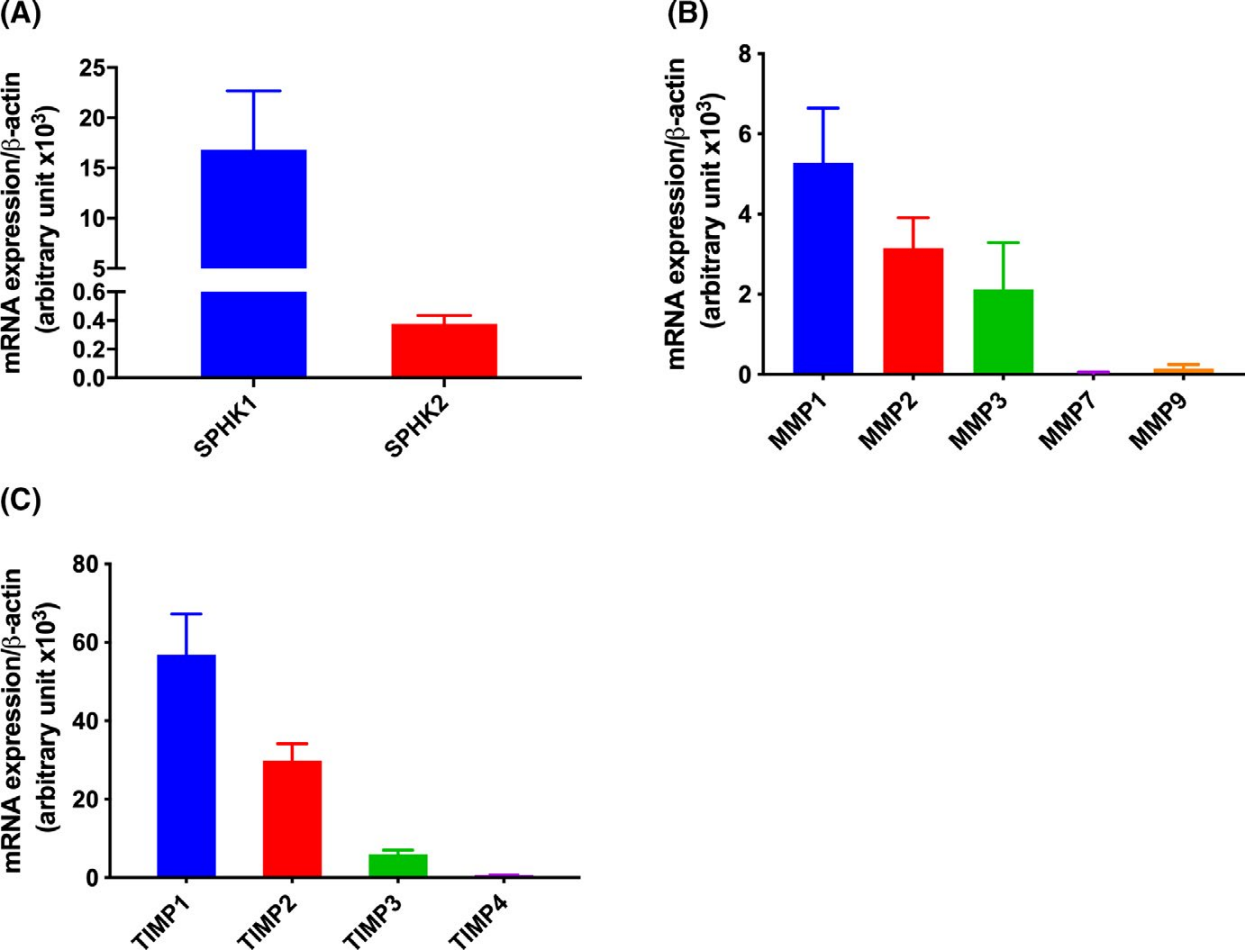
Expression analysis of *SPHKs*, *MMPs*, and *TIMPs* in HTR‐8/SVneo cells. The expressions of different genes were measured by real‐time PCR. Bar diagrams show the expression of A) *SPHK1* and *SPHK2*; B) matrix metalloproteinases *MMP1*, *MMP2*, *MMP3*, *MMP7,* and *MMP9*; C) tissue inhibitors of matrix metalloproteinases *TIMP1*, *TIMP2*, and *TIMP3*. All the genes except *MMP7* and *MMP9* were expressed in HTR‐8/SVneo cells. The data are means + SD of three independent experiments with different passages of HTR‐8/SVneo cells

To examine *SPHK1* and *SPHK2* genes' expression in HTR‐8/SVneo cells, we measured the abundance of mRNA by real‐time PCR. We found that *SPHK1* is highly expressed (~45‐fold) as compared to *SPHK2* in these cells (Figure 1A). These data suggest a functional role of SPHK1 in EVT cells.

Matrix remodeling proteins are crucial for cell migration and placental development. We first analyzed the basal expression of these genes in an EVT cell line model, HTR‐8/SVneo cells. We could find the expression of *MMP1*, *MMP2,* and *MMP3* genes in HTR‐8/SVneo cells, but *MMP7* and *MMP9* were not in the detectable range (Figure 1B). Further, we checked the expression of *TIMPs* and found that all the *TIMP* genes (*TIMP1‐3*) except *TIMP4* were expressed in EVT. Expression of *TIMP3* was ~10‐fold lower than that of *TIMP1* (Figure 1C). The differential expression of *MMPs* and *TIMPs* suggested a cell‐specific role of these genes in EVT.

### 3.2 Extracellular S1P gets transported in HTR‐8/SVneo cells

**FIGURE 2:**
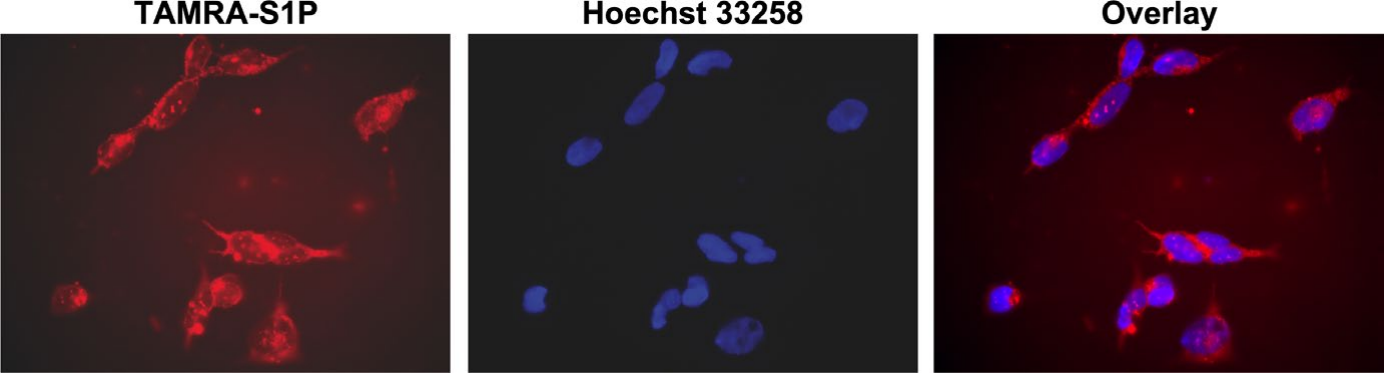
Extracellular S1P transport in the cytoplasm of HTR‐8/SVneo cells. A, Cells were treated with TAMRA‐S1P (1 µM, red, left panel) for 1 h, washed with PBS, and then nuclei were stained with Hoechst 33258 (blue, middle panel). The overlay is shown in the right panel. TAMRA‐S1P was able to enter the cells within 30 minutes

Previous studies showed that a high concentration of extracellular S1P significantly increases the intracellular level by 7‐fold.^20^ To confirm that exogenous S1P can enter the cells, we incubated the cells with fluorescently tagged S1P (TAMRA‐S1P, 1 µM). In agreement with previous studies,^20^, ^32^ we could observe that TAMRA‐S1P was transported into the cells (Figure 2). The data show that S1P is transported into the cells and it might have a role independent of receptor activation.

### 3.3 SPHKs‐dependent S1P regulates the expression of *MMP1* and *MMP3* in HTR‐8/SVneo cells

**FIGURE 3:**
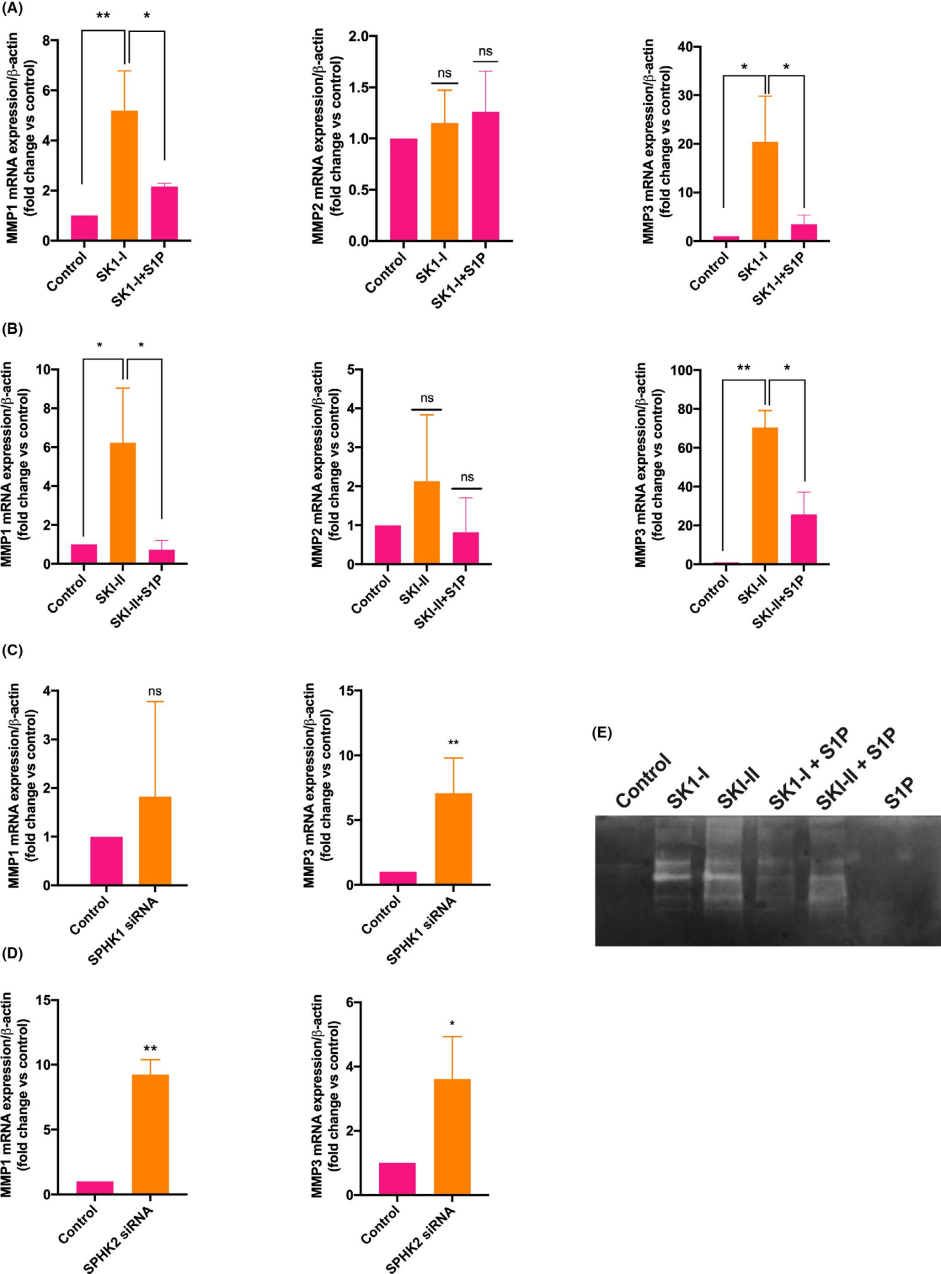
SPHKs regulate the expression of *MMP1* and *MMP3* genes. HTR‐8/SVneo cells were treated with solvent (control), specific SPHK1 inhibitor SK1‐I (10 µM), SPHK 1 and 2 inhibitor, SKI‐II (10 µM), and then the expressions of different genes were measured by real‐time PCR. A & B, Bar diagrams show the effect of A) SK1‐I and B) SKI‐II on the expression of *MMP1*, *MMP2,* and *MMP3* genes. The expression of *MMP1* (left panel) and *MMP3* (right panel) but not *MMP2* (middle panel) was significantly upregulated after the treatment of SK1‐I and SKI‐II. The upregulation of these genes was reduced by treating the cells with S1P (10 µM). C) *SPHK1* and D) *SPHK2* genes were knocked down in HTR‐8/SVneo cells using *SPHK1* and *SPHK2* specific siRNA treatment. The bar diagram shows the effect of gene knockdown on the expression of *MMP1* and *MMP3* genes. E) Gelatin zymography gel shows the activity of MMPs in control and cells treated with S1P, SK1‐I, and SKI‐II. The activity of MMPs are visible as clear areas (bands) on the gel, indicating where the gelatin is digested. The data are means + SD of three independent experiments with different passages of HTR‐8/SVneo cells. *P < .05; **P < .01; ***P < .001

To examine whether SPHKs regulate the gene expression of MMPs, we used the specific SPHK1 inhibitor SK1‐I,^33^, ^34^ and SKI‐II (inhibitor of both isoforms).^35^, ^36^ We performed concentration and time course for SPHKs inhibitors and S1P and found that SK1‐I and SKI‐II showed maximum effect at 10 µM concentration after 24 hours of treatment (Figure  [Link], [Link], [Link]). Cells were, therefore, treated with S1P (10 µM), SK1‐I (10µM), and SKI‐II (10µM) for 24 hours in subsequent experiments. We found that the expression of *MMP1* (~5‐ fold; *P* < .001; Figure 3A left panel) and *MMP3* (~20‐fold; *P* < .01; Figure 3A right panel) was significantly upregulated after the treatment of cells with SK1‐I for 24 hours. Similarly, the expression of *MMP1* (~6.6‐fold; *P* < .01; Figure 3B left panel) and *MMP3* (~70‐fold; *P* < .001; Figure 3B right panel) was significantly upregulated after the treatment of cells with SKI‐II inhibitor. The effect of SK1‐I and SKI‐II inhibitors on *MMP2* gene expression was not found to be significant (Figure 3A,B, middle panels). The expression of MMPs was confirmed by gelatin zymography. The activity of MMPs could not be observed in control cells and cells treated with S1P for 24 hours   and significantly enhanced in cells treated with SK1‐I and SKI‐II (Figure 3E). The enhanced activity was reversed upon treatment with S1P (Figure 3E). These data suggest that SPHK1 or both the isoforms play a crucial role in regulating MMP1 and MMP3 expression.

We asked whether the formation of intracellular S1P by SPHKs has a role in regulating *MMPs* gene expression. To examine the effect of S1P on the expression of *MMP*s, cells treated with SK1‐I and SKI‐II were co‐incubated with 10 µM S1P, as extracellular S1P can be internalized by the cells. We found that the upregulation of *MMP1*, *MMP3* was significantly reduced in SK1‐I or SKI‐II‐treated cells (Figure 3A,B). These data suggest that SPHKs‐dependent intracellular S1P regulates the gene expression of *MMP1* and *MMP3*.

To identify the specific role of SPHK1 and SPHK2 in regulating the expression of MMPs, we knocked down *SPHK1* and *SPHK2* genes in HTR‐8/SVneo cells using specific *SPHK1* and *SPHK2* siRNAs. A set of three different siRNAs were used for each SPHK isoform. Only one siRNA against the *SPHK1* gene could inhibit the expression by ~65% (Figure S1A). All three siRNAs against *SPHK2* could knock down the *SPHK2* gene by ~92% (Figure S1B). In contrast to the effect of SKI‐1, *MMP3* (~7‐fold; *P* < .01) but not *MMP1* gene was significantly upregulated after the knockdown of SPHK1 (Figure 3C). This observation might be due to the partial knockdown of *SPHK1* by siRNA. Additionally, *MMP1* (~9‐fold; *P* < .01) and *MMP3* (~3.6‐fold; *P* < .05) were significantly upregulated after the knockdown of the *SPHK2* gene (Figure 3D). These data indicate that both SPHK1 and SPHK2 regulate the expression of *MMP1* and *MMP3*.

### 3.4 SPHKs regulate the expression of the *TIMP3* in HTR‐8/SVneo cells

**FIGURE 4:**
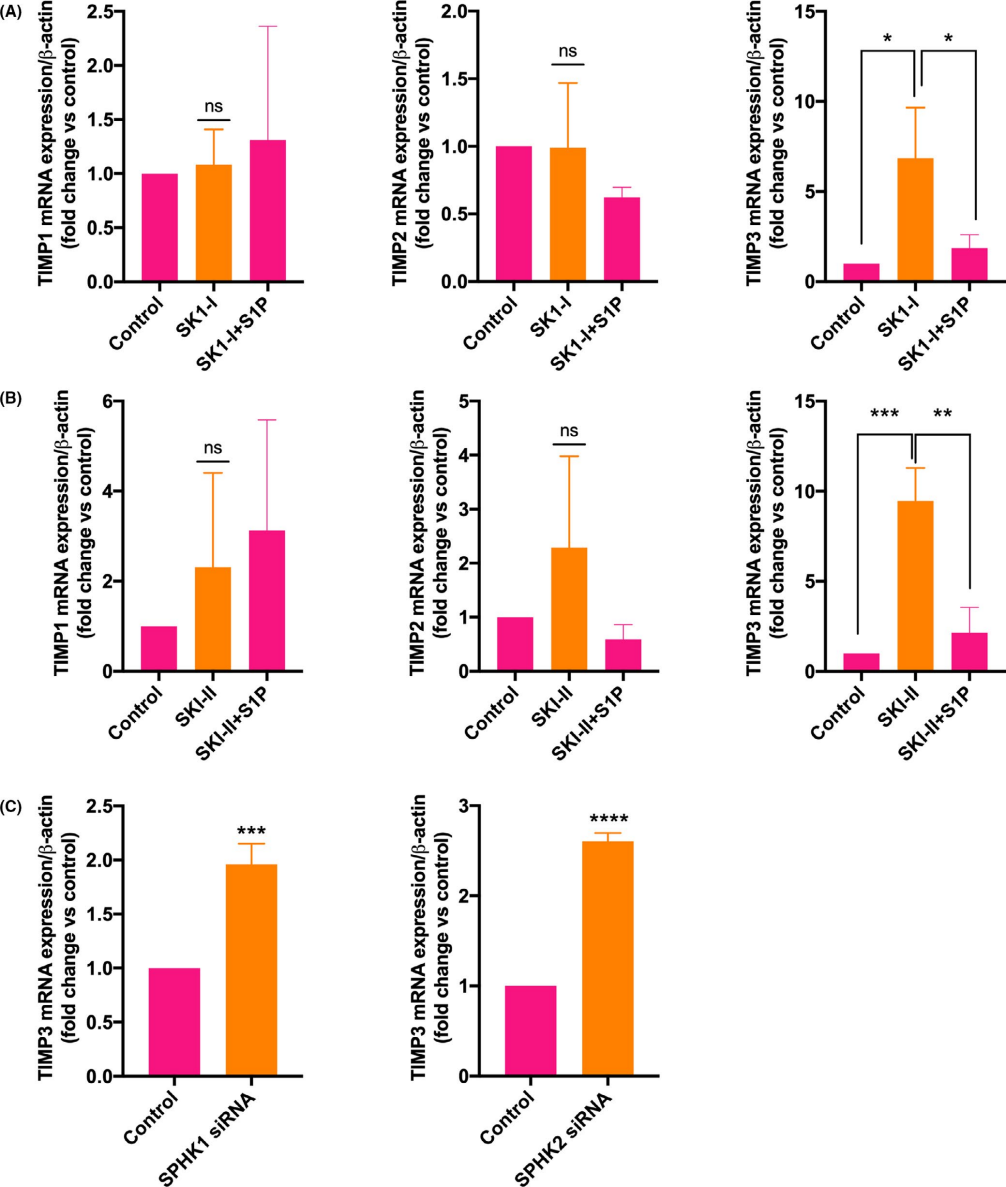
SPHKs regulate the expression of the *TIMP3* gene. HTR‐8/SVneo cells were treated with specific SPHK1 inhibitor SK1‐I (10 µM), SPHK 1 and 2 inhibitor SKI‐II (10 µM), and the expressions of *TIMPs* were measured by real‐time PCR. A) and B) Bar diagrams show the effect of A) SK1‐I and B) SKI‐II on the expression of *TIMP1*, *TIMP2,* and *TIMP3*. The expression of *TIMP3* (right panel) but not that of *TIMP1* and *TIMP2* (left and middle panels, respectively) was significantly upregulated with SK1‐I and SKI‐II treatment that was reduced after S1P (10 µM) treatment. C) Bar diagrams show the effect of *SPHK1* and *SPHK2* knockdown on the expression of *TIMP3*. The data are means + SD of three independent experiments with different passages of HTR‐8/SVneo cells. *P < .05; **P < .01; ***P < .001; ****P< .0001

Co‐expression of MMPs and TIMPs has been demonstrated in trophoblast cells.^6^ We asked whether SPHK/S1P axis also regulates the expression of *TIMP* *s* . When the cells were treated with SK1‐I for 24 hours, the expression of the *TIMP3* (~6.8‐fold; *P* < .01; Figure 4A right panel) was highly upregulated while *TIMP1* and *TIMP2* were not affected by SPHK1 inhibition (Figure 4A left and middle panels). Additionally, treatment of cells with SKI‐II could significantly induce the expression of *TIMP3* gene expression (~9.4‐fold; *P* < .0001; Figure 4B right panel) was significantly upregulated within 24 hours, while other two genes were not affected (Figure 4B left and middle panel). To further validate our data, we knocked down the *SPHK1* and *SPHK2* in HTR‐8/SVneo cells. The expression of the *TIMP3* was enhanced by ~2‐fold (*P* < .001) and by ~2.6‐fold (*P* < .0001) by knockdown of *SPHK1* and *SPHK2*, respectively (Figure 4C). These data indicate that SPHK1 and SPHK2 regulate the expression of *TIMP3*.

To determine the role of intracellular S1P on the expression of *TIMPs*, cells treated with SK1‐I and SKI‐II were incubated with 10 µM S1P. We found that the upregulation of *TIMP3* was significantly reduced in SK1‐I or SKI‐II‐treated cells (Figure 4A,B). These data suggest that SPHKs ‐dependent intracellular S1P regulates the gene expression of *TIMP3*.

### 3.5 S1P alone doesn't regulate the basal expression of *MMPs* and *TIMPs* in HTR‐8/SVneo cells

**FIGURE 5:**
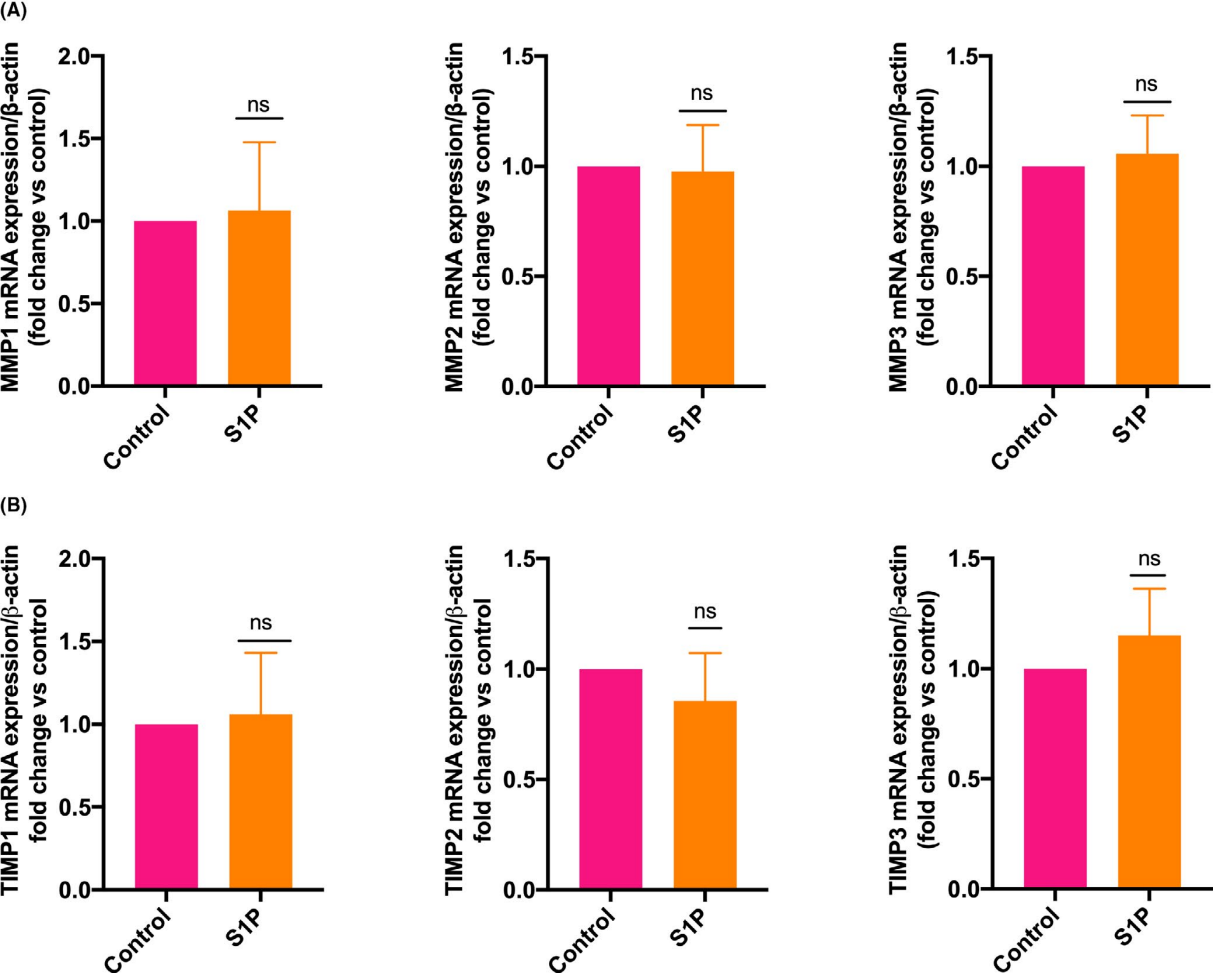
Extracellular S1P does not control the expression of *MMPs* and *TIMPs* expression. HTR‐8/SVneo cells were treated with solvent (control), S1P (10 µM), and the expressions of *MMP*s and *TIMPs* were measured by real‐time PCR. A) Bar diagrams show that extracellular S1P did not regulate the expression of *MMP1*, *MMP2,* and *MMP3*. B) Bar diagrams show that extracellular S1P did not regulate the expression of *TIMP1*, *TIMP2,* and *TIMP3*. The data are means + SD of three independent experiments with different passages of HTR‐8/SVneo cells. ns, not significant

To examine whether extracellular S1P regulates the gene expression of MMPs and TIMPs, we treated the cells with S1P (10 µM). We found that S1P did not significantly affect the expression of *MMPs* and *TIMPs* after treating the cells with S1P for 24 hours (Figure 5A,B). These data suggest that extracellular S1P did not control the basal expression of these genes. If the concentration of intracellular S1P is lower than the threshold, the expression of these genes is upregulated.

## 4 DISCUSSION

Trophoblast cells regulate the matrix remodeling via the secretion of various MMPs and TIMPs spatially and temporally. In this study, we evaluated the role of SPHKs and S1P in regulating *MMPs/TIMPs* expression in EVT cells. We could show that *MMP1*, *MMP2,* and *MMP3* genes were highly expressed in HTR‐8/SVneo cells. Expression of *MMP7* and *MMP9* was lesser as compared to the other genes in EVT cells. Our data are consistent with a previous report in which *MMP2* expression was observed in EVT cells and *MMP9* mainly in villous cytotrophoblast.^37^ Another study showed that *MMP2* is highly expressed in early trophoblast (6‐8 weeks) while *MMP9* is highly expressed in late first‐trimester trophoblasts (9‐12 weeks).^38^ *MMP1*, *MMP3,* and *MMP7* were differentially expressed in trophoblasts throughout the pregnancy.^10^ It suggests that the expression of a specific MMP depends on the trophoblasts' type and pregnancy stage. *TIMP1‐3* but not *TIMP4* was expressed in EVT cells.

In contrast, a study showed that all four TIMPs are expressed in cytotrophoblast cells.^9^ It was shown that TIMP2 was highly expressed in trophoblasts.^39^ Contrary to this, TIMP1 expression was increased from week 6 to 9 in cytotrophoblast while TIMP2 was undetectable.^11^ These studies suggest that differential regulation of TIMPs might regulate the migration and invasion of EVTs.

We showed at gene expression level for the first time that both the isoforms, *SPHK1* (~45‐fold of *SPHK2*) and *SPHK2*, were expressed in EVT cells. SPHK1 was downregulated in term placentae and term chorionic villous explants from patients with preeclampsia, suggesting a role of SPHK1 in trophoblast cell functions and preeclampsia.^40^ S1P induces the expression of MMP2 in endothelial cells,^41^ MMP7 in hepatocellular carcinoma cells,^42^ and MMP9 in breast cancer cells.^43^ In contrast, we could not find any change in the expression of *MMPs* and *TIMPs* after the treatment of HTR‐8/SVneo cells with S1P. Brocklyn et al. showed that intracellular S1P regulates apoptosis independent of S1P receptor 1.^32^ These data suggest that extracellular S1P does not control the expression of *MMPs* and *TIMPs* in EVT, and intracellular S1P might regulate the expression of these genes.

Various studies showed that SPHK1 promotes MMP2 and MMP9 expression in fibroblast‐like synoviocytes and colon carcinoma RKO cells.^24^, ^25^ Interestingly, we found that SPHK1 and SPHK2 negatively regulate the gene expression of *MMP1*, *MMP3,* and *TIMP3* in HTR‐8/SVneo cells. The expression of *MMP2, TIMP1, and TIMP2* was not affected significantly after the inhibition of SPHK1 and SPHK2. Together with our finding, we suggest that SPHKs differentially regulate the expression of MMPs and TIMPs family proteins in a cell type‐specific manner. The primary role of SPHKs is to phosphorylate sphingosine and produce intracellular S1P.^15^ These data suggest that SPHKs regulate the expression of these genes either independent or dependent on intracellular S1P. A study showed that activation of SPHK1 mediates the upregulation of TIMP1 in human fibroblast cells, and the intracellular level of S1P was increased in SPHK1 overexpressed cells.^28^ Previously, it was shown that the addition of exogenous S1P enhanced its intracellular level.^20^, ^32^ In agreement with the previous data, we could show that S1P could enter the cells by adding fluorescently labeled S1P to the cell culture medium. Intracellular S1P binds with PPARγ and PG1β complex and regulates the expression of various genes in endothelial cells.^19^ Intracellular S1P synthesized by SPHK2 acts as HDAC inhibitor and enhances the expression of *c‐fos* and cyclin‐dependent kinase inhibitor p21.^17^ Another study showed that SPHK1 and S1P bind with TRAF2 and regulate NF‐κB activation.^20^ Surprisingly, in this study, we showed that the addition of exogenous S1P inhibits the upregulation of *MMP1, MMP3,* and *TIMP3* , indicating a new role of intracellular S1P. These data suggest that SPHKs‐dependent intracellular S1P levels might act as a negative feedback switch and regulate the expression of *MMP1*, *MMP3,* and *TIMP3*, specifically in EVTs.

S1P and SPHK1 play an essential role in various cellular processes, including cell migration and invasion in different cell types.^44^ S1P inhibits cell migration in C2C12 myoblasts via the S1PR2 receptor.^45^ S1P promotes EVT cell invasion through MMP2 using S1P/S1PR1 signaling.^23^ In this study, we could not observe the effect of S1P on EVT cell migration (Figure S2). A study showed that S1P attenuated the migration and thus outgrowth of EVT from the first‐trimester placental explant.^46^ These contrasting results might be due to the cell type‐specific signaling. In preeclampsia, HDAC9 was downregulated, and knockdown of HDAC9 upregulated the expression of *TIMP3* in HTR‐8/SVneo cells.^27^ Poor EVT cell migration and invasion were observed in preeclampsia.^47^ Overall, we propose that SPHKs‐dependent regulation of TIMP3 might play an essential role in preeclampsia.

**FIGURE 6:**
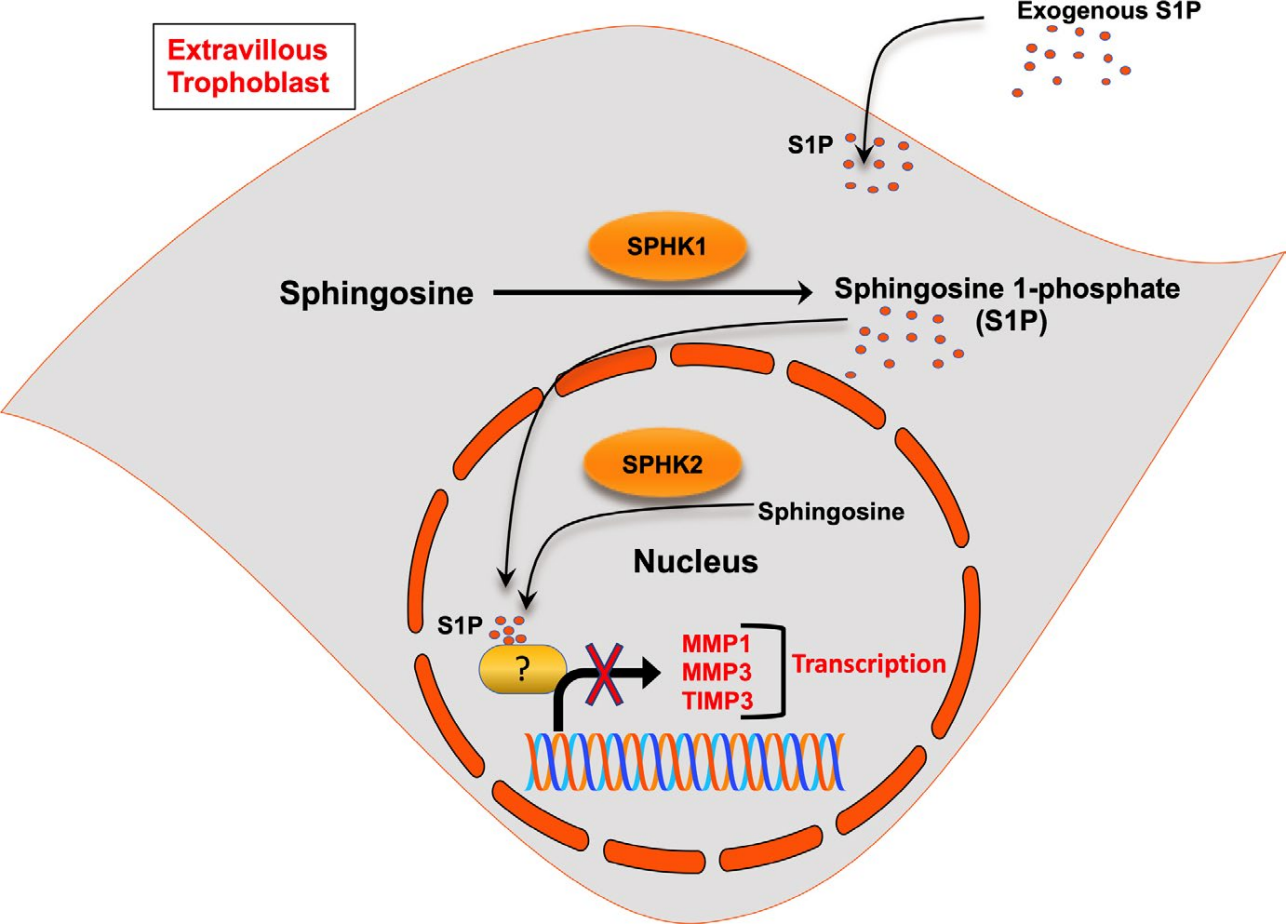
Proposed model of the role of intracellular S1P. The cartoon shows the role of intracellular S1P in *MMP1*, *MMP3,* and *TIMP3* expression and regulation

In conclusion, we found that the level of intracellular S1P acts as a controlling switch for *MMP1*, *MMP3,* and *TIMP3* expression in EVT cells, suggesting a new role of intracellular S1P in ECM remodeling (Figure 6). Together with previous studies, we propose that downregulation of SPHKs in pregnancy disorders, such as preeclampsia^40^ decreases the intracellular S1P level leading to the activation of *TIMP3* ^27^ and *MMP1,* and *MMP3*.

## DISCLOSURES

*Conflict of interest*: The authors declare that they have no conflict of interest. *Human/Animal rights*: This article does not contain any studies with human and animal subjects performed by any authors.

## Supporting information

Fig S1

Fig S2

Fig S3

Fig S4

Fig S5

Table S1
