# Supplementary material for: Sphingosine kinases negatively regulate the expression of matrix metalloproteases (MMP1 and MMP3) and their inhibitor TIMP3 genes via sphingosine 1‐phosphate in extravillous trophoblasts
Source: Reprod Med Biol. 2021 Mar 22;20(3):267–76. doi: 10.1002/rmb2.12379 (PMC8254167; doi:10.1002/rmb2.12379)
Supplement: Supplementary file 1 — Fig S1 [file RMB2-20-267-s003.pdf]

Figure 1S

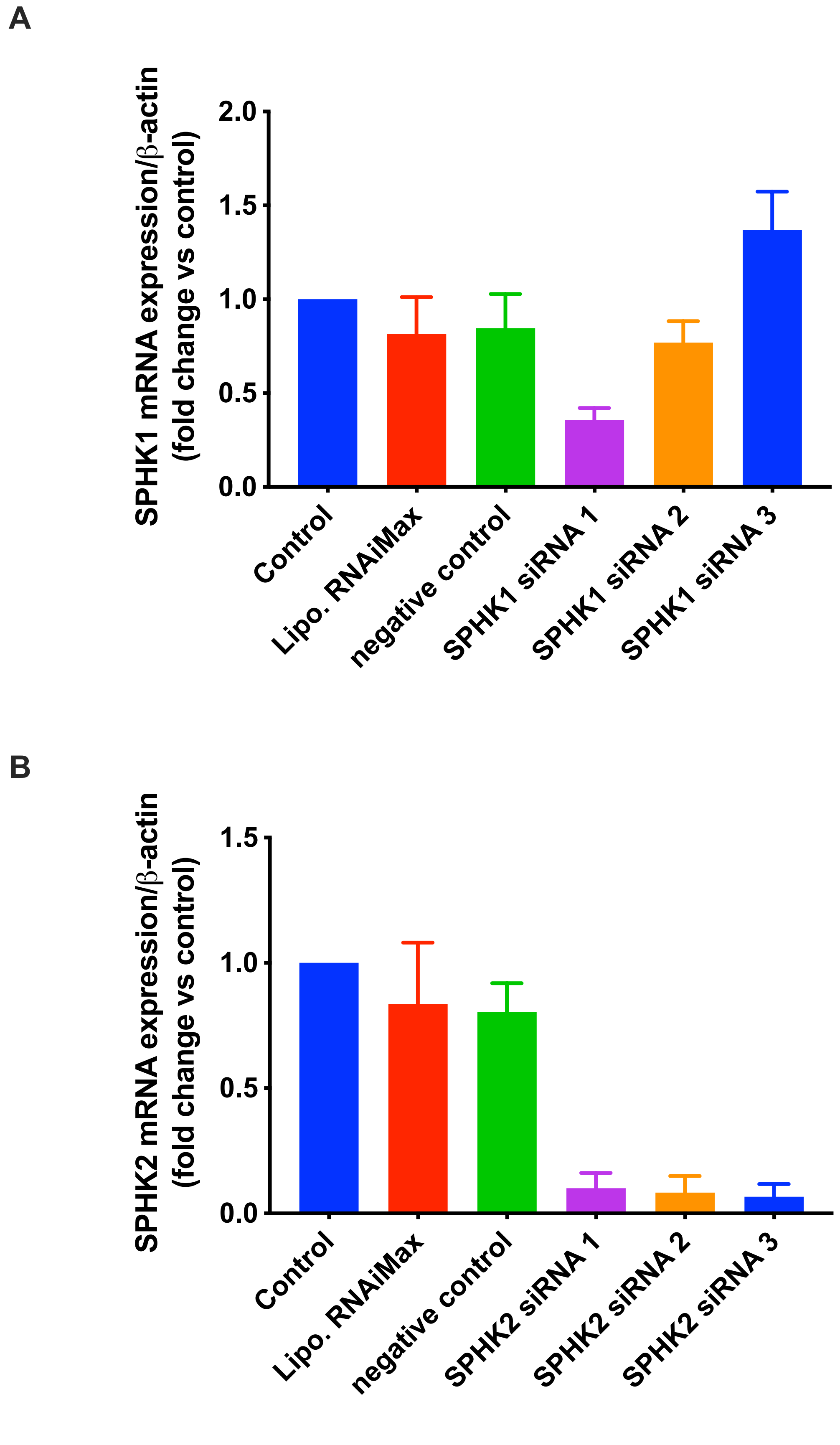

Figure S1: Knockdown efficiency of *SPHK1* and *SPHK2* specific siRNAs.

Cells were transfected with *SPHK1* or *SPHK2* specific siRNA as described in materials and methods. The expression of A) *SPHK1* and B) *SPHK2* genes were measured by real-time PCR. Scrambled siRNA was used as negative control
