# Supplementary material for: Sphingosine kinases negatively regulate the expression of matrix metalloproteases (MMP1 and MMP3) and their inhibitor TIMP3 genes via sphingosine 1‐phosphate in extravillous trophoblasts
Source: Reprod Med Biol. 2021 Mar 22;20(3):267–76. doi: 10.1002/rmb2.12379 (PMC8254167; doi:10.1002/rmb2.12379)
Supplement: Supplementary file 2 — Fig S2 [file RMB2-20-267-s005.docx]

**Method**

**Migration assay**

Cell migration assay was performed as described previously.^1^ In brief, cells were seeded in a 24‑well plate and the assay was performed with control cells and S1P- and SPHKs inhibitors-treated cells. After 24 hours of seeding, a scratch was created in the cell layer to generate a cell gap. The medium was changed to a serum-starved medium and cells were treated as described above. Cell migration for closing the gap was visualized using a Leica DMI6000 inverted microscope. The images were captured at a 10‑fold magnification.

**Result: SPHKs and S1P did not regulate HTR-8/SVneo cell migration**

We asked whether SPHKs and S1P regulate the HTR 8/SVneo cell migration. We could not observe any effect of the treatment of S1P and that of the specific SPHK1 inhibitor; SK1-I, on the migration of HTR-8/SVneo cells (Figure S2). SPHKs inhibitor SKI-II could significantly reduce the cell migration of HTR-8/SVneo cells (Figure S2). SKI-II severely affected the cell morphology within 24 hours of treatment, which was observed as reduced size and round or elongated shape (Figure S2).


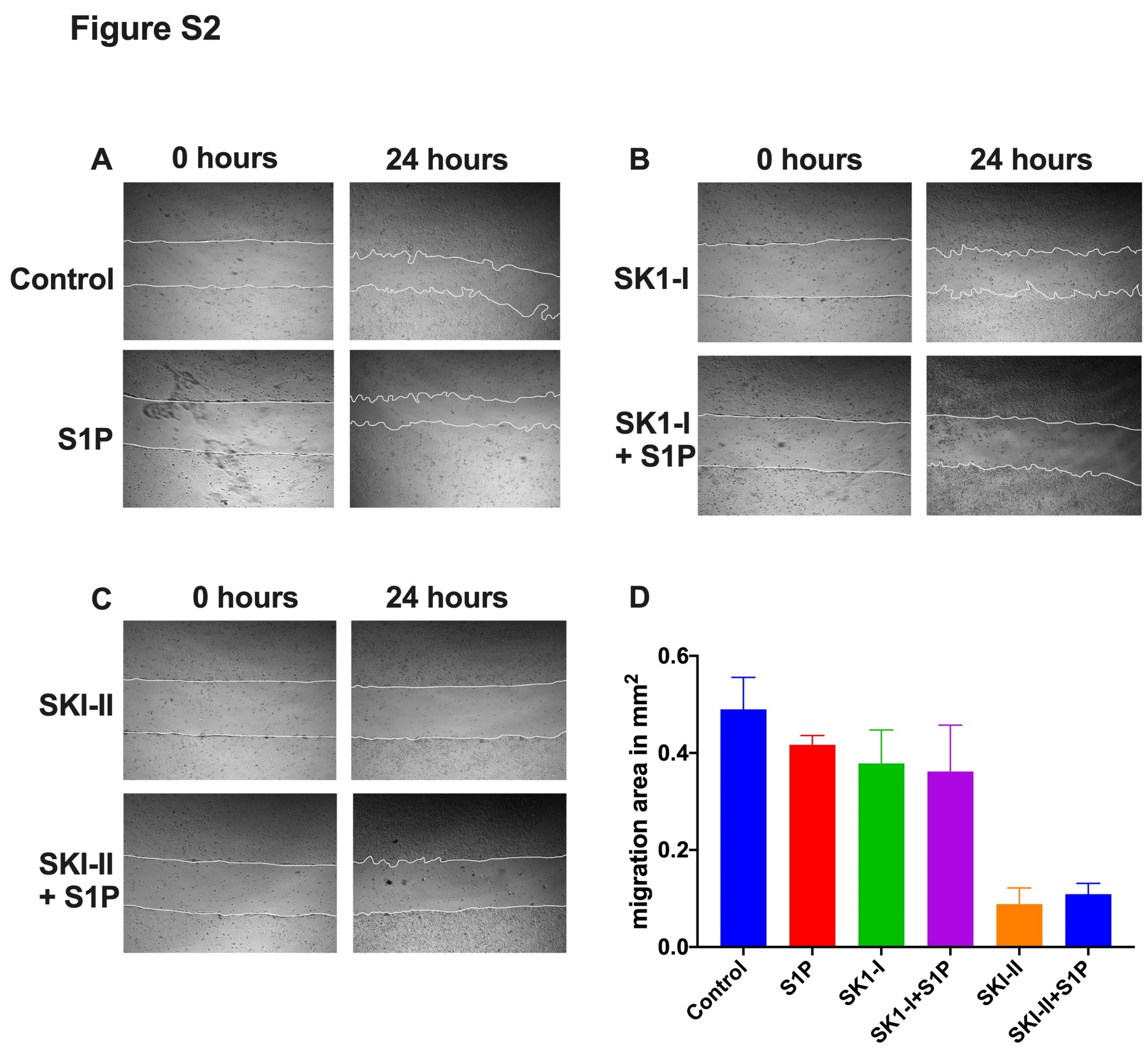


Figure S2: **SPHKs and S1P did not regulate HTR-8/SVneo cell migration**

HTR‑8/SVneo cells were plated in a 24‑well plate and grown in a factors-reduced medium. Cells were then treated with S1P (10 µM), SK1-I (10 µM) or SKI-II (10 µM). A wound scratch assay was performed for 24h to analyze the migration behavior of the cells. S1P or SK1-I could not affect the migration of HTR-8/SVneo cells, whereas SKI-II reduced the migration and severely affected the cell morphology.

1. Brünnert D, Shekhawat I, Chahar KR, et al. Thrombin stimulates gene expression and secretion of IL-11 via protease-activated receptor-1 and regulates extravillous trophoblast cell migration. *J Reprod Immunol.* 2019;132:35-41.
