# Supplementary material for: Sphingosine kinases negatively regulate the expression of matrix metalloproteases (MMP1 and MMP3) and their inhibitor TIMP3 genes via sphingosine 1‐phosphate in extravillous trophoblasts
Source: Reprod Med Biol. 2021 Mar 22;20(3):267–76. doi: 10.1002/rmb2.12379 (PMC8254167; doi:10.1002/rmb2.12379)
Supplement: Supplementary file 3 — Fig S3 [file RMB2-20-267-s001.docx]

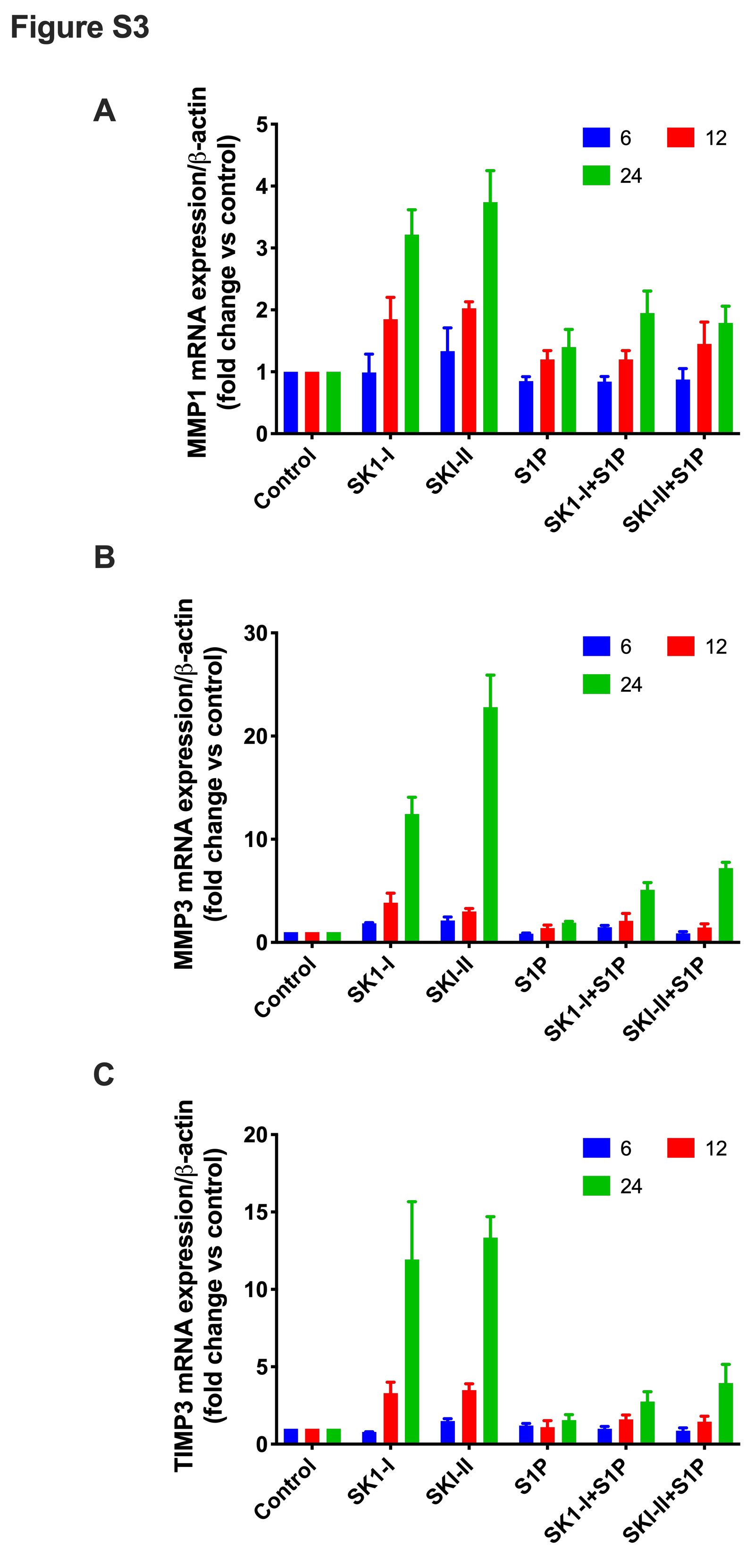


**Figure S3: Expression analysis of *MMP1*, *MMP3* and *TIMP3* genes at different time points.** HTR-8/SVneo cells were treated with solvent (control), specific SPHK1 inhibitor SK1-I (10 µM), SPHK 1 and 2 inhibitor, SKI-II (10 µM), and then the expressions of *MMP1*, *MMP3* and *TIMP3* genes were measured by real-time PCR at different time intervals. We found that these genes were maximally upregulated at 24 hours.
