# Supplementary material for: Sphingosine kinases negatively regulate the expression of matrix metalloproteases (MMP1 and MMP3) and their inhibitor TIMP3 genes via sphingosine 1‐phosphate in extravillous trophoblasts
Source: Reprod Med Biol. 2021 Mar 22;20(3):267–76. doi: 10.1002/rmb2.12379 (PMC8254167; doi:10.1002/rmb2.12379)
Supplement: Supplementary file 5 — Fig S5 [file RMB2-20-267-s002.docx]

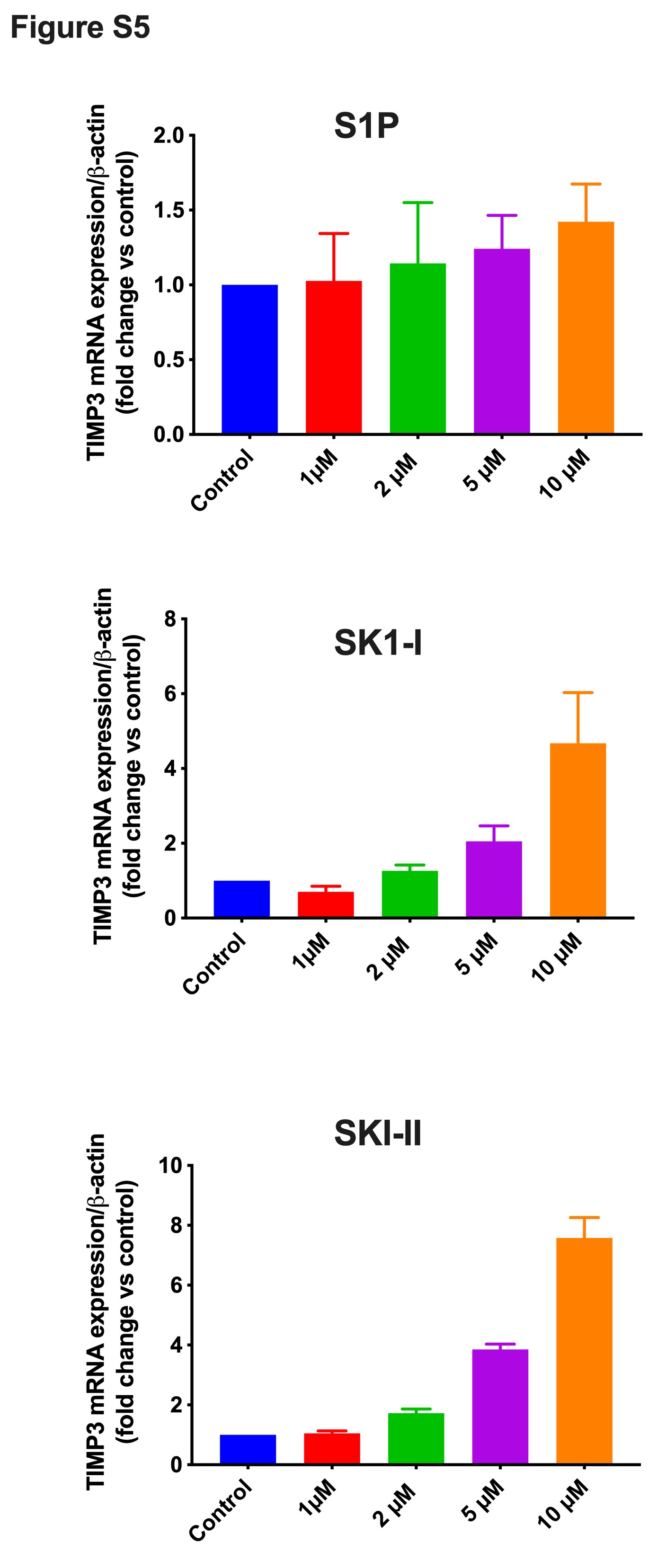


**Figure S5: Expression analysis of *TIMP3* after treating the cells with different concentrations of S1P and SPHKs inhibitors.** HTR-8/SVneo cells were treated with solvent (control), different concentrations of S1P, specific SPHK1 inhibitor SK1-I, and SPHK 1 and 2 inhibitor, SKI-II for 24 hours, and then the expressions of *TIMP3* gene were measured by real-time PCR. We found that *TIMP3* was maximally upregulated at 10 µM concentration of both inhibitors while S1P couldn’t show any effect.
