# Supplementary material for: Sphingosine kinases negatively regulate the expression of matrix metalloproteases (MMP1 and MMP3) and their inhibitor TIMP3 genes via sphingosine 1‐phosphate in extravillous trophoblasts
Source: Reprod Med Biol. 2021 Mar 22;20(3):267–76. doi: 10.1002/rmb2.12379 (PMC8254167; doi:10.1002/rmb2.12379)
Supplement: Supplementary file 6 — Table S1 [file RMB2-20-267-s006.docx]

Supplementary table 1: List of primers used in the study

| Gene Name | Gene ID | Forward Sequence | Reverse Sequence |
| --- | --- | --- | --- |
| ACTB | NM_001101.3 | GCACTCTTCCAGCCTTCCTT | AATGCCAGGGTACATGGTGG |
| SPHK1 | NM_021972.3 | TCACCCATGAACCTGCTGTC | CCCAGACGCCGATACTTCTC |
| SPHK2 | NM_020126.4 | AAGCTGGGCTGTCCTTCAAC | GTTCAGCACCTCATGGAGCA |
| S1PR1 | NM_001400.4 | GCAGCTCGGTCTCTGACTAC | GAACACCACCGAGGTCAGTT |
| S1PR2 | NM_004230.3 | TCATCCTCTGTTGCGCCATT | ACAGGTACATTGCCGAGTGG |
| S1PR3 | NM_005226.3 | AGGCTCAGTGGTTCATCGTG | GTTGCAGACCAGACGGAAGA |
| TIMP1 | NM_003254.2 | GCATCCTGTTGTTGCTGTGG | GAACTTGGCCCTGATGACGA |
| TIMP2 | NM_003255.4 | GCTGGACGTTGGAGGAAAGA | CAGGCTCTTCTTCTGGGTGG |
| TIMP3 | NM_000362.4 | ACCTGCCTTGCTTTGTGACT | AGGCGTAGTGTTTGGACTGG |
| MMP1 | NM_002421.3 | ATGTGGAGTGCCTGATGTGG | CTCTTGGCAAATCTGGCGTG |
| MMP2 | NM_004530.5 | CCTGCAAGTTTCCATTCCGC | CTTCTTGTCGCGGTCGTAGT |
| MMP3 | NM_002422.4 | ATCCTACTGTTGCTGTGCGT | GGTTCATGCTGGTGTCCTCA |
| MMP7 | NM_002423.4 | CATGATTGGCTTTGCGCGAG | CGTCCAGCGTTCATCCTCAT |
| MMP9 | NM_004994.2 | GGCGCTCATGTACCCTATGT | TTCAGGGCGAGGACCATAGA |
